# Emergent Intervention Criterias for Controlling Sever Bleeding after Percutaneous Nephrolithotomy

**DOI:** 10.1155/2013/760272

**Published:** 2013-07-28

**Authors:** Ural Oguz, Berkan Resorlu, Mirze Bayindir, Tolga Sahin, Omer Faruk Bozkurt, Ali Unsal

**Affiliations:** ^1^Department of Urology, Kecioren Training and Research Hospital, Ankara, Turkey; ^2^Department of Urology, Gazi University School of Medicine, Ankara, Turkey

## Abstract

*Objectives*. To determine when emergent intervention for bleeding after percutaneous nephrolithotomy (PCNL) is required. *Methods*. We reviewed analysis data of 850 patients who had undergone PCNL in our center. Blood transfusion was needed for 60 (7%) patients during and/or after surgery. We routinely performed followup of the urine output per hour, blood pressure, and hemoglobin levels after PCNL. Five (0.6%) of them had severe bleeding that emergent intervention was needed. *Results*. The mean age of the 5 patients who had emergent surgery due to severe bleeding was 42.2 (19–56) years. Mean duration of surgery was 44.75 (25–65) minutes. Mean stone size was 27 (15–38) mm. Mean decrease of hemoglobin was 4.8 (3.4–5.8) ng/dL, and unit of transfused blood was 4.4 (3–6). Mean blood pH was 7.21. There were metabolic acidosis and anuria/oliguria in all these patients. One of 5 patients suffered from cardiopulmonary arrest because of massive bleeding four hours after the PCNL, and despite cardiac resuscitation, he died. Hemorrhaging was controlled by open surgery in the other 4 patients. Two patients experienced cardiac arrest during the open surgery but they responded to cardiac resuscitation. There were no metabolic asidosis and anuria/oliguria, and bleeding was managed only with blood transfusion for the other 55 patients. *Conclusion*. Severe bleeding after PCNL is rare and can be mortal. If metabolic asidosis and anuria/oliguria accompanied the drop of hemoglobin, emergent surgical intervention should be performed because vascular collapse may follow, and it may be too difficult to stabilise the patient.

## 1. Introduction

Urolithiasis is a common disease, and the prevalence of this disease is increasing everyday [1]. Percutaneous nephrolithotomy (PCNL) is an effective and common treatment technique for especially large and complex renal calculi. Although PCNL is a common procedure, it can be associated with some mortal or morbid complications such as septicemia, severe bleeding, and pleural or colonic injury. With this study, we documented patients who needed blood transfusion and patients who had renal hemorrhaging requiring emergent intervention after PCNL. We aimed to answer questions including “how long conservative therapy for bleeding after PCNL takes?” and “when emergent intervention should be performed?” To our knowledge, this is the first paper to investigate this topic regarding PCNL.

## 2. Material and Methods 

### 2.1. Patients

We performed a retrospective analysis of 850 patients who underwent PCNL in the urology department of our institute. Before the operation, patients were evaluated with abdominal X-ray, ultrasonography, intravenous urography (IVU), and/or computerized tomography. Blood samples were analyzed for serum biochemistry, complete blood count, and coagulation tests. Urinalysis and urine culture were also analyzed. All patients were instructed not to use antithrombotic or antiagregan agents like aspirin for at least 1 week before the procedure. 

### 2.2. Surgical Technique

The PCNL operation was performed following the standard procedure, starting with the insertion of a ureteral catheter in the lithotomy position. The surgeon continued the operation with the patient in a supine or prone position. Percutaneous access was performed under fluoroscopic guidance by the urologist. Tract dilation was achieved by Amplatz, metal, or balloon dilators. 11-F, 15.9-F, 22-F, or 26-F rigid nephroscopes were used. Stone fragmentation was performed using pneumatic and ultrasound energy. In some cases, a holmium laser was used with a flexible nephroscope for stones which were impossible to reach with a rigid nephroscope. Mostly, a nephrostomy tube was placed at the end of the operation and removed after 1 to 3 days. Tubeless procedure was performed for selected patients. Patients were discharged when there was no leakage detected in the nephrostomy tract. 

### 2.3. Postoperative Followup

In our clinic, we check the hemoglobin immediately following PCNL and again 24 hours later in all patients. If necessary, checks were performed at more frequent intervals. We also routinely perform followup of the urine output per hour after PCNL. If anuria or oliguria is detected, we focus on the vital signs like blood pressure, pulse, and we check the blood count and blood gas analysis for metabolic acidosis. Anuria/oliguria and decrease of hemoglobin could disappear with the fluid resuscitation and blood transfusion or could not. But in case of metabolic asidosis associated with anuria/oliguria and decrease of hemoglobin, emergent surgical intervention was needed. 

## 3. Results

Blood transfusion was necessary for 60 (7%) patients. Five of them had severe bleeding that emergent open exploration was needed. Bleeding was self-limited, and blood transfusion was enough for other 55 patients.

For those 55 patients, male/female ratio was 3/4. Mean patient age was 45 (1–76) years; median patient age was 49 years. Twenty-six patients had the operation on the right side, while 29 had the operation on the left side. Seven (11%) patients had more than 1 access, while others had 1 access. The number of upper pole access was 9, middle pole access was 11, and lower pole access was 42. Mean operation time was 84.91 (25–170) minutes. Mean stone size was 39.87 (15–99) mm. Mean decrease of hemoglobin was 3.98 (1–7.6) g/dL, and mean blood transfusion ratio was 1.77 (0.25–7) units. Mean hospitalisation time was 5.4 (3–14) days (Table 1).

Nineteen of the 60 patients who needed blood transfusions had anuria or oliguria with a decrease of hemoglobin. Of these 19 patients, metabolic asidosis, however, was detected in only 5. 

Of these 5 patients who needed emergent intervention, one patient died, and four of them underwent renal exploration following the first 24 h of PCNL. The patient who died due to severe bleeding had paraplegia because of a road accident and had the history of open nephrolithotomy surgery. The other four patients had no comorbidity before the operation. Mean time between PCNL and intervention was 13 hours. Mean duration of PCNL surgery was 44.75 (25–65) minutes. Mean stone size was 27 (15–38) mm. All had lower pole access, and Amplats dilators were used for the tract dilation in all patients. Four patients had the operation on the left side, while one had the operation on the right side. Mean drop of hemoglobin for these 5 patients was 4.8 (3.4–5.8) g/dL. Mean blood transfusion ratio was 4.4 (3–6) units. Mean blood pH was 7.21. They all had metabolic asidosis and anuria/oliguria with drop of hemoglobin. Of these patients, one suffered from cardiopulmonary arrest because of massive hemorrhaging four hours after the operation. There was not enough time for intervention, and despite cardiac resuscitation, the patient had died. In the other 4 patients, hemorrhaging was controlled by open surgery because immediate embolization was not available. Two of these patients suffered from cardiac arrest during the open surgery, but they responded to cardiac resuscitation. Characteristics of these 5 patients are shown in Table 2.

The next finding after a decrease of hemoglobin was anuria or oliguria. However, if there was a metabolic asidosis in the blood gas analysis, transfusion was not enough to keep the patients stable. So, from anuria/oliguria, situation progressed to metabolic asidosis. Another important finding for these patients was the appearance of vascular collapse, detected after metabolic asidosis.

Because we did not have an angiography unit in our institute, we performed open surgery for this emergent situation. We observed that there was not any injury to vessels and bleeding was from the dilation tract. We controlled the bleeding by primer suturation in all patients. There was a hematoma around the kidney in retroperitoneum, and the patients became stable dramatically just after removing the hematoma. 

We detected arteriovenous fistula in 3 (0.3%) patients and pseudoaneurysm in 2 (0.2%) patients after the procedure as a late bleeding, and these patients were treated with elective embolization successfully. These late bleedings were not life-threatening.

## 4. Discussion

Urolithiasis is a common disease, and percutaneous nephrolithotomy (PCNL) is an effective treatment for especially large and complex renal calculi. PCNL has become a common procedure since it was described in 1976 [2]. Although PCNL is a common procedure, it can be associated with some mortal or morbit complications. There are some studies investigating the prediction of morbidity and mortality of this surgery [3–5]. The complication rate of PCNL is up to 83%, but they are generally minor complications [6]. Renal hemorrhaging requiring intervention is a rare complication of PCNL, and its frequency is 0.6–1.4% [6]. In our series, 1.17% of the patients needed intervention for renal hemorrhaging, and half of them (0.58%) had emergent surgical intervention.

Renal hemorrhage is generally associated with the nephrostomy tract, operative time, method of tract dilatation and access guidance, number of tract, renal parenchymal thickness, absence of hydronephrosis, intraoperative puncture time, size and location of stones, upper calyceal access, extensive angulation with rigid nephroscope, diabetes mellitus, and the experience of surgeon [7–12]. During PCNL a grade IV renal injury occurs, and bleeding can appear during every step of the operation. Decrease of hemoglobin can be seen after all PCNL operations; however, this is generally self-limited because of the restrictive effect of the Gerota's fascia and retroperitoneum. Therefore, bleeding is often controlled by conservative measures like monitoring the level of hemoglobin and vital signs with fluid resuscitation therapy or sometimes a blood transfusion. That is why PCNL is also a safe procedure for selected patients using anticoagulant therapy [13].

In the literature, blood transfusion rate was reported as 4.9% to 9.5% [4, 14]. In our series, blood transfusion rate is 7%. Arteriovenous fistula was detected in 3 (0.3%) patients pseudoaneurysm in 2 (0.2%) patients in 7 to 15 days after the PCNL, and they were treated by embolisation. Severe bleeding occurred in 5 patients. One patient died because of severe hemorrhaging immediately after the operation. Four patients needed emergent open surgical exploration after the operation. The main triads for requiring emergency intervention were decrease of hemoglobin, anuria/oliguria, and metabolic asidosis.

Anuria or oliguria with a decrease of hemoglobin was detected in 19 of 60 patients (31.6%) who had blood transfusions. Of 19 patients, metabolic asidosis was detected in 5, and these 5 patients were those who needed emergent surgical exploration. Metabolic asidosis was not detected in the other patients. Vasculary collapse is the next step after metabolic asidosis. 

Bleeding can be an important complication as it has been described in our study. It is important to make decisions for emergent surgery to control bleeding after PCNL. One patient died, and 2 patients had cardiac arrest after the surgery. We determined that surgical intervention for patients who had the triad mentioned above is essential, and if intervention is postponed, the risk of vascular collapse is extreme.

## 5. Conclusion

Although PCNL is a safe procedure for the treatment of renal calculus, it sometimes results in some complications. Bleeding after PCNL is generally self-limited and can be treated with conservative measurements. However, it is important to determine the time for emergent intervention. If the decrease of hemoglobin is associated with anuria/oliguria and metabolic asidosis, emergent surgical intervention should be performed before vasculary collapse.

## Figures and Tables

**Table 1:** Characteristics of patients who had bleeding and blood transfusion.

	Gender (M/F)	Age (year)	Mean stone size (mm)	Operation time (minute)	Access (lower/middle /upper pole)	Mean hemoglobin (preop/postop)	Mean blood transfusion (unit)	Anuria /oliguria (*n*)	Mean blood pH	Hospitalisation time (day)
Patients treated with blood transfusion (*n*: 55)	24/31	45 (1–76)	39.87 (15–99)	84.91 (25–120)	42/11/9	12.98/9.03	1.77 (0.25–7)	14	7.36 (7.33–7.42)	5.3 (3–14)

Patients needed emergent intervention (*n*: 5)	5/—	42.2 (19–56)	27 (15–38)	44.75 (25–65)	5/—/—	13.3/8.7	4.4 (3–6)	5	7.21 (7.20–7.25)	7.75 (5–10)

**Table 2:** Characteristics of patients who needed emergent open surgical exploration for bleeding after percutaneous nephrolithotomy.

Gender	Age (year)	Access	Time between PCNL and intervention (h)	Blood pH	Hemoglobin (preop/postop)	Eritrosit transfusion (unit)	Hospitalisation (day)
M	41	Subcostal lower pole	10	7.25	12.2/8.8	6	5
M	56	Subcostal lower pole	6	7.23	12.3/7.7	4	10
M	40	Subcostal lower pole	12	7.20	14.2/9.8	3	9
M	19	Subcostal lower pole	24	7.20	14.4/8.6	5	7
M	55	Subcostal lower pole	4	7.20	13.4/8.6	4	ex
